# Impact of topology in foliated quantum Einstein gravity

**DOI:** 10.1140/epjc/s10052-017-5046-8

**Published:** 2017-07-24

**Authors:** W. B. Houthoff, A. Kurov, F. Saueressig

**Affiliations:** 1 Institute for Mathematics, Astrophysics and Particle Physics (IMAPP), Radboud University Nijmegen, 6525 AJ Nijmegen, The Netherlands; 2 Department of Theoretical Physics, Moscow State University, Moscow, Russia

**Keywords:** Quantum gravity, Asymptotic safety, Renormalization group

## Abstract

We use a functional renormalization group equation tailored to the Arnowitt–Deser–Misner formulation of gravity to study the scale dependence of Newton’s coupling and the cosmological constant on a background spacetime with topology $S^1 \times S^d$. The resulting beta functions possess a non-trivial renormalization group fixed point, which may provide the high-energy completion of the theory through the asymptotic safety mechanism. The fixed point is robust with respect to changing the parametrization of the metric fluctuations and regulator scheme. The phase diagrams show that this fixed point is connected to a classical regime through a crossover. In addition the flow may exhibit a regime of “gravitational instability”, modifying the theory in the deep infrared. Our work complements earlier studies of the gravitational renormalization group flow on a background topology $S^1 \times T^d$ (Biemans et al. Phys Rev D 95:086013, 2017, Biemans et al. arXiv:1702.06539, 2017) and establishes that the flow is essentially independent of the background topology.

## Introduction and motivation

Asymptotic Safety, first suggested by Weinberg [3, 4], constitutes a mechanism for constructing a consistent and predictive quantum theory for gravity within the framework of quantum field theory. A central goal of the program is to give meaning to the path integral over (Euclidean) metrics:

1 $$\begin{aligned} \mathcal{Z}= \int \mathcal{D}\hat{g}\, \exp (-S[\hat{g}]), \end{aligned}$$

with $S[\hat{g}]$ a suitable, diffeomorphism invariant action functional. This task can be addressed along various ways [5], e.g. by applying continuum renormalization group methods or discrete Monte Carlo techniques.

Within the causal dynamical triangulations (CDT) program, reviewed in [6], the action entering the partition function (1) is taken as the Einstein–Hilbert action and the partition sum is taken on background topologies $S^1 \times S^3$ [7–9] or on topologies of the form $S^1 \times T^3$ [10]. $\mathcal{Z}$ is then evaluated on piecewise linear geometries constructed from elementary simplices.^1^ The simplices provide a lattice regularization, making the partition sum finite. Removing the regulator by taking the continuum limit then requires a second order phase transition where the correlation length diverges. For CDT a candidate for such a phase transition has been identified in [15, 16], also see [17–19] for related investigations. Moreover, random walks on CDT spacetimes exhibit manifold-like behavior for long diffusion time, indicating the presence of a classical phase [20–22]. These features are typically attributed to the presence of a causal structure associated with the $S^1$-factor in the topology which allows building up spacetime as a stack of spatial slices.

A second route towards asymptotic safety, reviewed in [23–28], converts the partition sum (1) into a functional renormalization group equation (FRGE) for the effective average action $\Gamma _k$ [29–31]. Starting from the pioneering work [32], this program has made significant progress in demonstrating that the asymptotic safety mechanism may lead to a viable quantum theory of gravity. In particular, the existence of a non-Gaussian fixed point (NGFP), which constitutes the key element in this program, has been demonstrated in a wide range of approximations [33–47]. Starting from [48–50] renormalization group flows which resolve the difference between the background and fluctuation fields have been constructed, e.g., in [51–61] while the role of the path-integral measure has recently been discussed in [62–65].^2^

Despite their common root given by the partition sum (1), a systematic link between results obtained within the CDT program and the FRGE approach is still missing. While the spectral dimension of the resulting quantum spacetimes have been compared in [73], little is known about the relation of the two formulations. On this basis, the present work devises an FRGE study which incorporates all the essential features underlying the Monte Carlo simulations carried out within CDT. The natural continuum analogue of the foliation structure imposed on the microscopic spacetimes studied within CDT is the Arnowitt–Deser–Misner (ADM) formulation reviewed, e.g., in [74]. In this formalism spacetime is built up from a stack of spatial hypersurfaces $\Sigma _\tau$ on which the time-variable $\tau$ is constant. These hypersurfaces are welded together such that they fill the entire spacetime. The resulting preferred “time” direction obtained in this way plays a similar role as the causal structure implemented in CDT.

An FRGE tailored to the ADM formalism has been constructed in [75, 76] and we will use this framework in the sequel.^3^ This construction makes manifest use of the background field formalism. Since most CDT simulations restrict the geometries contributing to (1) to be of topology $S^1 \times S^2$ or $S^1 \times S^3$, we evaluate the flow equation on a background geometry given by $S^1 \times S^d$ where the (intrinsic) curvature of $S^d$ is a free parameter. Moreover, the flow is projected onto Einstein–Hilbert action which provides the weight of the partition sum (1) in the CDT framework.

Our work is complementary to the recent investigation [1, 2] in the sense that it uses a different background topology. It also provides a detailed analysis on how the flow is influenced by integrating over different classes of spatial fluctuations and under a change of the regulator scheme. As a main result, we find that all cases studied in this paper admit a NGFP suitable for Asymptotic Safety. The phase diagrams obtained from integrating the flow equations are strikingly similar to the ones found for background topology $S^1 \times T^d$ [1, 2]. In particular, we show that there are specific combinations of parameterizing the metric fluctuations and regulating the flow equation which realizes the double-fixed point scenario found in [1] *in four spacetime dimensions*. In this case the RG trajectory realized by Nature, as described in [80], is well defined on all length scales. The mechanism underlying the completion of the RG trajectories in the deep infrared is closely related to the proposal of “erasing the cosmological constant through a gravitational instability”, recently made in [81].

The rest of the work is organized as follows. Section 2 introduces the essential elements of the ADM formalism together with the corresponding FRGE. The beta functions governing the flow of Newton’s coupling and the cosmological constant are constructed in Sect. 3 and their properties are analyzed in Sect. 4. We close with a brief discussion of our findings in Sect. 5. Technical details as regards the background geometry, the structure of the flow equation, and the evaluation of the operator traces are provided in Appendix A, Appendix B, and Appendix C, respectively.

## Renormalization group flows in the ADM formalism

Our construction of the gravitational renormalization group (RG) flow is based on the FRGE for the effective average action [29–31] tailored to the Arnowitt–Deser–Misner (ADM) formulation [75, 76]. This section summarizes the central points of the construction.

### Parametrization of the fluctuation fields

The ADM formalism decomposes the spacetime metric $g_{\mu \nu }$ into a lapse function $N(\tau ,y)$, a shift vector $N_i(\tau ,y)$ and a metric $\sigma _{ij}(\tau ,y)$. The later measures distances on the spatial slices $\Sigma _\tau$ defined by $\tau = \mathrm{const}$. For Euclidean signature this decomposition is given by

2 $$\begin{aligned} \mathrm{{d}}s^2= & {} \, g_{\mu \nu } \, \mathrm{{d}}x^\mu \mathrm{{d}}x^\nu \nonumber \\= & {} \, N^2 \mathrm{{d}}\tau ^2 + \sigma _{ij} \, (\mathrm{{d}}y^i + N^i \mathrm{{d}} \tau ) (\mathrm{{d}}y^j + N^j \mathrm{{d}} \tau ). \end{aligned}$$

At the level of the metric tensor, this entails

3 $$\begin{aligned} g_{\mu \nu } =&\, \left( \begin{array}{cc} N^2 + N_i N^i \; \; &{} \; \; N_j \\ N_i &{} \sigma _{ij} \end{array} \right) , \nonumber \\ g^{\mu \nu } =&\, \left( \begin{array}{cc} \frac{1}{N^{2}} \; \; &{} \; \; - \frac{N^j}{ N^{2}} \\ - \frac{N^i}{ N^{2}} \; \; &{} \; \; \sigma ^{ij} + \, \frac{ N^i \, N^j}{ N^{2}} \end{array} \right) . \end{aligned}$$

An infinitesimal coordinate transformation acts on the spacetime metric via $\delta g_{\alpha \beta } = \mathcal{L}_v \, g_{\alpha \beta }$ where $\mathcal{L}_v$ is the Lie derivative. This transformation induces the transformation law for the component fields

4 $$\begin{aligned} \delta N= & {} \,\partial _\tau (f N ) + \zeta ^k \partial _k N - N N^i\partial _i f, \nonumber \\ \delta N_i= & {} \, \partial _\tau ( N_i f) + \zeta ^k\partial _k N_i + N_k\partial _i\zeta ^k \nonumber \\&+ \, \sigma _{ki}\partial _\tau \zeta ^k + N_k N^k\partial _i f + N^2\partial _i f \, , \nonumber \\ \delta \sigma _{ij}= & {} \, f \, \partial _\tau \sigma _{ij} + \zeta ^k \, \partial _k \sigma _{ij} + \sigma _{jk} \, \partial _i\zeta ^k + \sigma _{ik} \, \partial _j\zeta ^k \nonumber \\&+\, N_j\, \partial _i f + N_i\partial _j f, \end{aligned}$$

where the vector $v^\alpha$ has been decomposed into a time-component *f* and a vector tangent to the spatial slice $\zeta ^i$.

The construction of the flow equation for the ADM formalism uses the background field method. The quantum fields $N, N_i, \sigma _{ij}$ are decomposed into a fixed (but arbitrary) background $\bar{N}, \bar{N}_i, \bar{\sigma }_{ij}$ and fluctuations around this background $\hat{N}, \hat{N}_i, \hat{\sigma }_{ij}$. For the lapse function and the shift vector, we resort to a linear split

5 $$\begin{aligned} N = \bar{N}+ \hat{N}, \quad N_i = \bar{N}_i + \hat{N}_i. \end{aligned}$$

The fluctuations of $\sigma _{ij}$ may be parametrized either through a linear or an exponential split

6 $$\begin{aligned} \text{ linear: } \quad&\sigma _{ij} = \bar{\sigma }_{ij} + \hat{\sigma }_{ij} \, , \nonumber \\ \text{ exponential: } \quad&\sigma _{ij} = \bar{\sigma }_{il} [e^{\hat{\sigma }}]^l{}_j. \end{aligned}$$

Here indices are raised and lowered with the background metric. Essentially, the choice of split (6) determines the type of fluctuations admissible in the construction: the exponential split guarantees that $\sigma _{ij}$ and $\bar{\sigma }_{ij}$ have the same signature while in the linear split the fluctuations may change the signature of $\sigma _{ij}$. The exponential split of the spatial metric in the ADM decomposition then has the same effect as in the covariant construction [39, 63–65]: in both cases fluctuations cannot change the signature of the metric.

At the level of the spacetime metric, the exponential split (6) ensures that the signature of the time direction and spatial metric remains unchanged, independently of the value of the fluctuation fields. This can be seen from computing the determinant of $g_{\mu \nu }$. Applying the method of Schur complements yields

7 $$\begin{aligned} \det ( g_{\mu \nu }) = N^2 \, \det ( \sigma _{ij}). \end{aligned}$$

Here $N^2$ is positive by construction and the exponential split ensures that $\sigma _{ij}$ has the same signature as $\bar{\sigma }_{ij}$. Thus the ADM formalism in the exponential parametrization constitutes a refinement of the standard exponential parametrization by restricting the quantum fluctuations to the set which conserves the signature of the spatial and time part of the spacetime metric independently.

In terms of practical computations, it is convenient to combine the linear and exponential splits according to

8 $$\begin{aligned} \sigma _{ij} \simeq \bar{\sigma }_{ij} + \hat{\sigma }_{ij} + \tfrac{\alpha }{2} \, \hat{\sigma }_{ik} \, \bar{\sigma }^{kl} \, \hat{\sigma }_{lj} + \cdots . \end{aligned}$$

The parameter $\alpha$ takes the value $\alpha = 0$ for the linear and $\alpha = 1$ for the exponential split. The dots represent terms containing cubic and higher powers of the fluctuation fields. Since these terms will not contribute to the present computation we refrain from giving their explicit structure at this stage.

### The functional renormalization group equation

The scale dependence of coupling constants can conveniently be obtained from the FRGE for the effective average action $\Gamma _k$ [29–32]. Besides the gravitational action, $\Gamma _k$ also contains suitable gauge-fixing and ghost terms

9 $$\begin{aligned} \Gamma _k[\hat{\chi }; \bar{\chi } ] = \Gamma _k^\mathrm{grav}[\hat{\chi }; \bar{\chi } ] + \Gamma _k^\mathrm{gauge-fixing}[\hat{\chi }; \bar{\chi } ] + \Gamma _k^\mathrm{ghost}[\hat{\chi }; \bar{\chi } ].\nonumber \\ \end{aligned}$$

Here $\hat{\chi }$ and $\bar{\chi }$ denote the collection of fluctuation fields and background fields, respectively. The central property of $\Gamma _k$ is that its dependence on the RG scale *k* is governed by the formally exact RG equation

10 $$\begin{aligned} k \partial _k \Gamma _k = \frac{1}{2} \, \mathrm{Tr} \left[ \left( \Gamma _k^{(2)} + \mathcal{R}_k \right) ^{-1} \, k \partial _k \mathcal{R}_k \right] \, . \end{aligned}$$

Here $\Gamma _k^{(2)}$ is the second variation of $\Gamma _k$ with respect to the fluctuation fields and the trace indicates an integration over loop momenta. The regulator $\mathcal{R}_k$ provides a *k*-dependent mass term for the fluctuation modes with momenta $p^2 \ll k^2$ and vanishes for $p^2 \gg k^2$. In the propagator $(\Gamma _k^{(2)} + \mathcal{R}_k )^{-1}$, the regulator suppresses the contribution of fluctuations with momenta $p^2 \ll k^2$ to the trace. The term $k \partial _k \mathcal{R}_k$ in the numerator ensures that fluctuations with $p^2 \gg k^2$ do not contribute to the trace. As a consequence the right-hand-side of Eq. (10) is finite. Moreover, the flow of $\Gamma _k$ is driven by fluctuations whose momenta are comparable to the RG scale *k*.

The FRGE realizes several welcome features. Firstly, vertices extracted from $\Gamma _k$ include quantum corrections resulting from integrating out fluctuations with momenta $p^2 \gtrsim k^2$. Thus $\Gamma _k$ provides a one-parameter family of effective descriptions of physics at the scale *k*. This realizes Wilson’s idea of renormalization. Secondly, the FRGE may be used to study the RG flow and phase diagram of a theory without specifying an initial or fundamental action. This feature is particularly relevant in the context of asymptotic safety where the fundamental action is unknown a priori and arises as a fixed point of the flow. Such fixed points may be visible already in relatively simple projections of the FRGE. In the next section, we will utilize this feature and make a specific ansatz for the effective average action (9) in order to study RG flow of Newton’s coupling and the cosmological constant in a setting tailored to CDT.

## Einstein–Hilbert truncation on $S^1 \times S^d$

We now use the FRGE (10) to construct the beta functions governing the flow of Newton’s coupling and the cosmological constant on a background topology $S^1 \times S^d$.

### Ansatz for the effective average action

We approximate the gravitational part of $\Gamma _k$ by the Euclidean Einstein–Hilbert action. In terms of the ADM fields the resulting action is given by

11 $$\begin{aligned} \Gamma _k^\mathrm{grav} = \tfrac{1}{16 \pi G_k} \int \! \! \mathrm{{d}}\tau \mathrm{{d}}^dy N \sqrt{\sigma }[ K_{ij} K^{ij} - K^2 - R + 2 \Lambda _k]. \end{aligned}$$

Here

12 $$\begin{aligned} K_{ij} \equiv \frac{1}{2 N} ( \partial _\tau \sigma _{ij} - D_i N_j - D_j N_i) \end{aligned}$$

denotes the extrinsic curvature, $K \equiv \sigma ^{ij} K_{ij}$ is its trace, and *R* is the intrinsic curvature constructed from $\sigma _{ij}$. The ansatz comprises two scale-dependent couplings, Newton’s coupling $G_k$ and the cosmological constant $\Lambda _k$.

At this stage, it is convenient to make an explicit choice for the background fields. In the present work, we will choose a class of backgrounds with topology $S^1 \times S^d$. Explicitly, we take

13 $$\begin{aligned} \bar{N}= 1, \quad \bar{N}_i = 0, \quad \bar{\sigma }_{ij}(\tau ,y) = \bar{\sigma }_{ij}^{S^d}(y) \end{aligned}$$

where $\bar{\sigma }_{ij}^{S^d}$ is a one-parameter family of metrics on the sphere $S^d$ with radius *r* and independent of $\tau$. The curvature tensors for this background geometry then satisfy

14 $$\begin{aligned} \bar{K}_{ij} = 0 \end{aligned}$$

and

15 $$\begin{aligned} \bar{R}_{ijkl} = \tfrac{\bar{R}}{d(d-1)} \left( \bar{\sigma }_{ik} \bar{\sigma }_{jl} - \bar{\sigma }_{il} \bar{\sigma }_{jk} \right) , \quad \bar{R}_{ij} = \tfrac{1}{d} \bar{\sigma }_{ij} \bar{R}, \end{aligned}$$

with $\bar{R}$ being constant. A key property of this background is the existence of a global Killing vector field $\partial _\tau$. This feature allows one to perform a Wick rotation without generating a complex background geometry. Denoting expressions in Euclidean and Lorentzian signature by subscripts *E* and *L*, the corresponding analytic continuation is given by

16 $$\begin{aligned} \tau _E \rightarrow - i \tau _L, \quad N^i_E \rightarrow i N^i_L. \end{aligned}$$

At this stage it is instructive to verify that this background is sufficient to disentangle the flow of $G_k$ and $\Lambda _k$. For this purpose, we take the *k*-derivative of the ansatz (11) and subsequently set the fluctuation fields to zero

17 $$\begin{aligned} \left. k \, \partial _k \Gamma _k^\mathrm{grav} \right| _{\hat{\chi } = 0} = k \partial _k \left( \frac{1}{16 \pi G_k} \int \mathrm{{d}}\tau \mathrm{{d}}^dy \sqrt{\bar{\sigma }} \left[ - \bar{R}+ 2 \Lambda _k \right] \right) .\nonumber \\ \end{aligned}$$

This indicates that on the background (13) the flow of Newton’s coupling can be constructed from the coefficients multiplying the intrinsic background curvature while the beta function for the cosmological constant is encoded in the volume terms appearing on the left- and the right-hand-side of the FRGE. Thus it suffices to keep track of these two terms in the following.

The gravitational part of the effective average action has to be complemented by a suitable gauge-fixing and ghost action. Following the strategy [1], we use the gauge freedom in such a way that all fluctuation fields including the lapse function and the shift vector acquire a relativistic dispersion relation. Moreover, terms which are of the form $E \sqrt{\delta ^{ij}p_ip_j}$ are consistently eliminated from the Hessian $\Gamma _k^{(2)}$. These two conditions actually fix the choice of gauge uniquely,

18 $$\begin{aligned} \Gamma _k^\mathrm{gf} = \frac{1}{32 \pi G_k} \int \mathrm{{d}}\tau \mathrm{{d}}^dy \sqrt{\bar{\sigma }} \, \left[ F^2 + F_i \, \bar{\sigma }^{ij} \, F_j \right] . \end{aligned}$$

The *F* and $F_i$ are linear in the fluctuation fields and read

19 $$\begin{aligned} F= & {} \left[ \partial _\tau \hat{N}+ \bar{D^i}\hat{N_i} - \tfrac{1}{2} \partial _\tau \hat{\sigma } \right] \nonumber \\ F_i= & {} \left[ \partial _\tau \hat{N_i}-\bar{D}_i\hat{N} - \tfrac{1}{2} \bar{D}_i\hat{\sigma } + \bar{D^j}\hat{\sigma }_{ij} \right] , \end{aligned}$$

where $\hat{\sigma }\equiv \bar{\sigma }^{ij} \hat{\sigma }_{ij}$ and $\bar{D}_i$ denotes the covariant derivative constructed from $\bar{\sigma }_{ij}$. The gauge fixing (19) can be derived by adapting the harmonic gauge condition to the specific class of backgrounds and specifying the parametrization of the fluctuation fields to the one generated by the ADM decomposition. The action of the Faddeev–Popov ghosts is then constructed in the standard way. It comprises one pair of scalar ghosts $\bar{c},c$ and one pair of spatial vector ghosts $\bar{b}^i, b_i$. Restricting to terms quadratic in the fluctuation fields, the action reads

20 $$\begin{aligned} \Gamma _k^\mathrm {gh}= \int \mathrm{{d}}\tau \mathrm{{d}}^dy \sqrt{\bar{\sigma }} \, \big [ \, \bar{c} \, \Delta \, c + \bar{b}^i \, \left( \Delta - \tfrac{\bar{R}}{d} \right) \, b_i \big ], \end{aligned}$$

where $\Delta \equiv - \partial _\tau ^2 - \bar{\sigma }^{ij} \bar{D}_i \bar{D}_j$ is the *D*-dimensional Laplace operator constructed from the background spacetime.

In order to compute the propagator $(\Gamma _k + \mathcal{R}_k)^{-1}$, it is useful to perform a transverse-traceless decomposition of the fluctuation fields which is adapted to the background. The shift vector is decomposed into a transverse vector $u_i$ and a scalar *B*

21 $$\begin{aligned} \hat{N}_i = u_i + \bar{D}_i B, \quad \bar{D}^i u_i = 0. \end{aligned}$$

For the fluctuations of the spatial metric, we resort to the standard transverse-traceless decomposition of a symmetric tensor,

22 $$\begin{aligned} \hat{\sigma }_{ij} = h_{ij} + \bar{D}_i v_j + \bar{D}_j v_i + \left( \bar{D}_i \bar{D}_j-\tfrac{1}{d}\, \bar{\sigma }_{ij} \, \bar{D}^2\right) \psi + \tfrac{1}{d} \, \bar{\sigma }_{ij}h \end{aligned}$$

where the component fields are subject to the constraints

23 $$\begin{aligned} \bar{D}^i h_{ij} = 0, \quad \bar{\sigma }^{ij} h_{ij} = 0, \quad \bar{D}^i v_i = 0, \end{aligned}$$

and $\bar{\sigma }^{ij} \hat{\sigma }_{ij} \equiv \hat{\sigma }= h$. The Jacobians coming from these decompositions are absorbed into the momentum-dependent field redefinition (B.16). The matrix elements of $\Gamma _k^{(2)}$ with respect to these component fields are computed in Appendix B and summarized in Table 4. The result shows that the field decomposition diagonalizes $\Gamma _k^{(2)}$ in field space, apart from the scalar sector containing the two fields $\hat{N}$ and *h*.

The final ingredient required in the evaluation of the FRGE is the regulator $\mathcal{R}_k$. On the background (13) the Hessian $\Gamma _k^{(2)}$ is matrix valued in field space. From Table 4 one finds that the typical matrix element has the structure

24 $$\begin{aligned} \left[ \Gamma _k^{(2)} \right] _{ab} = \left( 32 \pi G_k\right) ^{-s} \, [\Delta + q \, \bar{R}+ \cdots ]_{ab} , \end{aligned}$$

where *s* takes the values 0, 1 and the dots represent a possible contribution from the cosmological constant. Moreover, *q* is a fixed, *d*-dependent numerical coefficient which depends on the field indices *a*, *b*. For example, the ghost action (20) leads to $s=0$ and $q_{\bar{c}c} = 0$ and $q_{\bar{b}b} = -1/d$. Based on the structure (24) there are two natural choices for a coarse-graining operator. Following the nomenclature introduced in [24], we define

25 $$\begin{aligned} \begin{array}{ll} \mathrm{Type I}: \qquad &{} \Box \equiv \Delta , \\ \mathrm{Type II}: \qquad &{} \Box \equiv \Delta + q \, \bar{R}. \end{array} \end{aligned}$$

For notational convenience, the two regularization schemes (25) are combined by introducing a parameter *r* via

26 $$\begin{aligned} \Box \equiv \Delta + r \, q \, \bar{R}. \end{aligned}$$

Setting $r=0$ or $r=1$ then corresponds to a regulator scheme of Type I and Type II, respectively. The matrix elements of $\mathcal{R}_k$ are then taken as operator-valued functions depending on the coarse-graining operator. Their explicit form can be obtained by the replacement rule

27 $$\begin{aligned} \Box \mapsto P_k \equiv \Box + R_k(\Box ) \, \end{aligned}$$

where $R_k(z)$ is a scalar profile function. The parametrization of the fluctuation fields combined with the specific choices for the coarse-graining procedure, which are considered in the following, are summarized in Table 1. At this stage, we have all the ingredients for evaluating the flow equation for the ansatz (11).

**Table 1:** Parameter sets used in analyzing the dynamics encoded in the beta functions (29). The value $q_\mathrm{off-diag}$ specifies the endomorphism in the $\hat{N}$–*h* sector, where the coarse-graining operator is then given by $\Box = \Delta + q_\mathrm{off-diag} \bar{R}$

Metric fluctuations	Regulator	$\alpha$	*r*	$q_\mathrm{off-diag}$
Linear	I	0	0	0
	II$_0$	0	1	0
	II$_1$	0	1	$\frac{d-2}{d}$
	II$_2$	0	1	$\frac{d-4}{d}$
Exponential	I	1	0	0
	II$_0$	1	1	0
	II$_1$	1	1	$\frac{d-2}{d}$
	II$_2$	1	1	$\frac{d-3}{d}$

We remark that for a Type II regulator scheme the coarse-graining operators $\Box$ are not necessarily positive semi-definite. Their explicit spectrum can be constructed from the eigenvalues of the Laplacian on the *d*-sphere listed, e.g., in [34]. In this way one finds that setting $r=1$ implies that $\Box$ has negative eigenvalues when acting on the constant $\psi$-mode and the two lowest eigenmodes in the *B*-sector if $d=3$. In addition the linear split combined with the II$_2$ regularization scheme leads to negative eigenvalues in the $\hat{N}$–*h* sector. The mode count for the vector ghosts is identical to the *B*–$u_i$ sector. The negative eigenvalues of $\Box$ occurring in the Type II case then suggest that the Type I regularization procedure may be preferred. The possibility for adjusting the spectrum of the coarse-graining operator by including a suitable endomorphism component may be used to implement conditions similar to the “equal lowest eigenvalue scheme” advocated in [62]. While it would be desirable to have a more complete understanding of the regulator dependence in the present case, we limit ourselves to the analysis of the cases introduced in Table 1.

We close this subsection with the following remark. In the companion paper [1] the setup (11) was used to construct the beta functions of $G_k$ and $\Lambda _k$ on a Euclidean Friedmann–Robertson–Walker background. In this case the background geometry is characterized by $\bar{R}= 0$ while one has a non-vanishing extrinsic curvature $\bar{K}_{ij}$. The beta function for the Newton coupling is then read off from the extrinsic curvature terms. At the level of classical general relativity the two incarnations of $G_k$ related to the extrinsic and extrinsic curvature terms coincide due to diffeomorphism invariance of the Einstein–Hilbert action. At the level of the FRGE it is expected that the two projection schemes may lead to (slightly) different results. Firstly, introduction of the regulator $\mathcal{R}_k$ may break the full diffeomorphism group to a subgroup so that the two projections actually construct the flow of two different coupling constants. Moreover, working on different backgrounds may result in different organization schemes for the fluctuation modes, indicating that modes integrated out at a certain scale *k* could be different in the two settings. The setting of this paper provides an ideal testing ground for obtaining a quantitative understanding of these effects.

### Beta functions

The beta functions governing the scale dependence of $G_k$ and $\Lambda _k$ are constructed in Appendix C. For conciseness, we limit ourselves to the expression obtained from setting the endomorphism piece in the scalar sector spanned by $\hat{N}$ and *h* to zero. Moreover, all threshold functions are evaluated with a Litim-type regulator (C.28). The result is conveniently expressed in terms of the dimensionless quantities

28 $$\begin{aligned} \eta \equiv (G_k)^{-1} \partial _t \, G_k, \quad \lambda _k \equiv \Lambda _k \, k^{-2}, \quad g_k \equiv G_k \, k^{d-1}, \end{aligned}$$

where $\eta$ is the anomalous dimension of Newton’s coupling. The scale dependence of the dimensionful couplings is then governed by the beta functions

29 $$\begin{aligned} k \partial _k g_k = \beta _g(g_k, \lambda _k), \quad k \partial _k \lambda _k = \beta _\lambda (g_k, \lambda _k). \end{aligned}$$

Defining

30 $$\begin{aligned} B_\mathrm{det} \equiv d-1 - (3d-2+ \alpha ) \lambda + 2 d \lambda ^2 \end{aligned}$$

one has

31 $$\begin{aligned} \beta _g= & {} (d-1+\eta ) \, g, \nonumber \\ \beta _\lambda= & {} \, (\eta - 2) \lambda + \tfrac{g}{(4\pi )^{(d-1)/2}} \Big [ -\tfrac{4 (d+1)}{\Gamma ((d+3)/2)}\nonumber \\&+\, \left( d + \tfrac{d^2+d-2}{2(1-(2-\alpha )\lambda )} + \tfrac{2(d-1) - (3d - 2 + \alpha ) \lambda }{B_\mathrm{det}} \right) \nonumber \\&\times \, \left( \tfrac{2}{\Gamma ((d+3)/2)} - \tfrac{\eta }{\Gamma ((d+5)/2)} \right) \Big ]. \end{aligned}$$

The anomalous dimension of Newton’s coupling takes the form

32 $$\begin{aligned} \eta = \frac{16 \pi g \, B_1(\lambda )}{(4\pi )^{(d+1)/2} + 16 \pi g \, B_2(\lambda )}. \end{aligned}$$

The functions $B_1$ and $B_2$ depend on $\lambda$ as well as the parameters $d,r,\alpha$. The terms appearing in these expressions are conveniently organized in terms of the contributions found in Appendix C.2,

33 $$\begin{aligned} B_{a} = B_{a,1} + B_{a,2} + B_{a,3} + B_{a,4} + B_{a,5}, \quad a =1,2. \end{aligned}$$

The explicit expressions for the building blocks are

34 $$\begin{aligned} B_{1,1}= & {} - \tfrac{d^3+3d^2+20d-6 + 6 r (d-1)^2}{12 \, d \, \Gamma ((d+3)/2)} + \tfrac{\delta _{2,d}}{d \, \Gamma ((d+1)/2)}, \nonumber \\ B_{1,2}= & {} \tfrac{d^4 - 15 d^2 - 22d + 12 + 3\left( d^3+3d^2+6d-4 \right) \delta _{2,d}}{12 \,d\, (d-1) \, (1 - (2-\alpha ) \lambda ) \, \Gamma ((d+1)/2)} \nonumber \\&-\, r \, \tfrac{ d^2 ( d-3) (1 + d) (2-\alpha ) + 4 d \, (3 - 2 \alpha ) - 4 \, (3 - \alpha ) }{4 \,d\, (d-1) \, (1 - (2-\alpha ) \lambda ) \, \Gamma ((d+1)/2)}, \nonumber \\ B_{1,3}= & {} (r-1) \, \tfrac{ d^2 (d^2-2d-3)(2-\alpha ) + 4 d (3 - 2 \alpha ) - 4 (3 - \alpha ) }{4 d (d-1) (1-(2-\alpha )\lambda )^2 \, \Gamma ((d+3)/2)}, \nonumber \\ B_{1,4}= & {} \tfrac{2(d-1) - (3d - 2 + \alpha ) \lambda }{6 \, B_\mathrm{det} \, \Gamma ((d+1)/2)}, \nonumber \\ B_{1,5}= & {} - \tfrac{(d-2)}{2d} \, \tfrac{ (d-1) ( 3 d -4 + \alpha ) -8d(d-1) \lambda + 2 d (3 d + \alpha ) \lambda ^2 }{ B_\mathrm{det}^2 \, \Gamma ((d+3)/2)}, \end{aligned}$$

and

35 $$\begin{aligned} B_{2,1} = \tfrac{d^3 + 3 d^2 + 6 d -6 + 6 r (d+1)^2}{24d \, \Gamma ((d+5)/2)} + \tfrac{\delta _{2,d}}{2 d \, \Gamma ((d+3)/2)}. \end{aligned}$$

The remaining coefficients in $B_2$ are proportional to their $B_1$ counterparts

36 $$\begin{aligned} B_{1,2}= & {} (d+1) B_{2,2}, \quad B_{1,3} = (d+1) B_{2,3}, \nonumber \\ B_{1,4}= & {} (d+3) B_{2,4}, \quad B_{1,5} = (d+3) B_{2,5}. \end{aligned}$$

This result completes the derivation of the beta functions for $g_k$ and $\lambda _k$ on the background topology $S^1 \times S^d$. Notably $\beta _\lambda$ is independent of the endomorphism parameter *r* but retains information on the parametrization of the metric fluctuations. For $\alpha = 0$, it agrees with the flow of the cosmological constant obtained on a $S^1 \times T^d$-background [2]. The beta functions (31) are the main result obtained in this section.

## Properties of the renormalization group flow

The beta functions derived in the previous section explicitly retain information on the parametrization of the fluctuation fields, encoded in the parameter $\alpha$, and the choice of regularization scheme, parametrized by *r*. Typically, these parameters have distinguished values. In the following subsection, we investigate how these choices affect the flow of $g_k$ and $\lambda _k$. Throughout the discussion we will limit ourselves mostly to the case $d=3$, corresponding to a four-dimensional spacetime.

### Fixed point structure and phase diagrams

The beta functions (29) constitute a system of autonomous coupled non-linear differential equations. In order to understand the dynamics of the system, it is useful to first determine its fixed points and singularity structure.

Singularities in the beta functions (31) can be traced back to two sources. First, there are loci in the *g*–$\lambda$ plane where the threshold functions diverge. The location of these lines depends on $\alpha$ and is independent of *r*. Evaluating the roots of Eq. (30), one finds

37 $$\begin{aligned} \alpha = 0: \quad&\lambda _1^\mathrm{sing} = \tfrac{1}{2}, \quad&\lambda _2^\mathrm{sing} = \tfrac{d-1}{d}, \nonumber \\ \alpha = 1: \quad&\lambda _1^\mathrm{sing} = \tfrac{d-1}{2d}, \quad&\lambda _2^\mathrm{sing} = 1. \end{aligned}$$

All singular lines are independent of *g* and located at $\lambda > 0$. Notably, the exponential parametrization moves the singular line $\lambda _1^\mathrm{sing}$ closer to the origin. The singularity $\lambda _1^\mathrm{sing}$ is the counterpart of the gravitational instability discussed in Ref. [81]. Besides these fixed singularities, there are also lines in the *g*–$\lambda$ plane where the anomalous dimension of Newton’s coupling diverges. In this case the denominator in Eq. (32) vanishes. Exploiting the $B_2(\lambda )$ is independent of *g*, this line is conveniently described by a parametrized curve obtained by equating the denominator of $\eta$ to zero and solving for $g^\mathrm{sing}$:

38 $$\begin{aligned} \eta ^\mathrm{sing}: \quad g^\mathrm{sing} = - \frac{2^{(d-3)} \, \pi ^{(d-1)/2}}{B_2(\lambda )}. \end{aligned}$$

The position of this singular line depends on the two parameters *r* and $\alpha$.

**Fig. 1:**
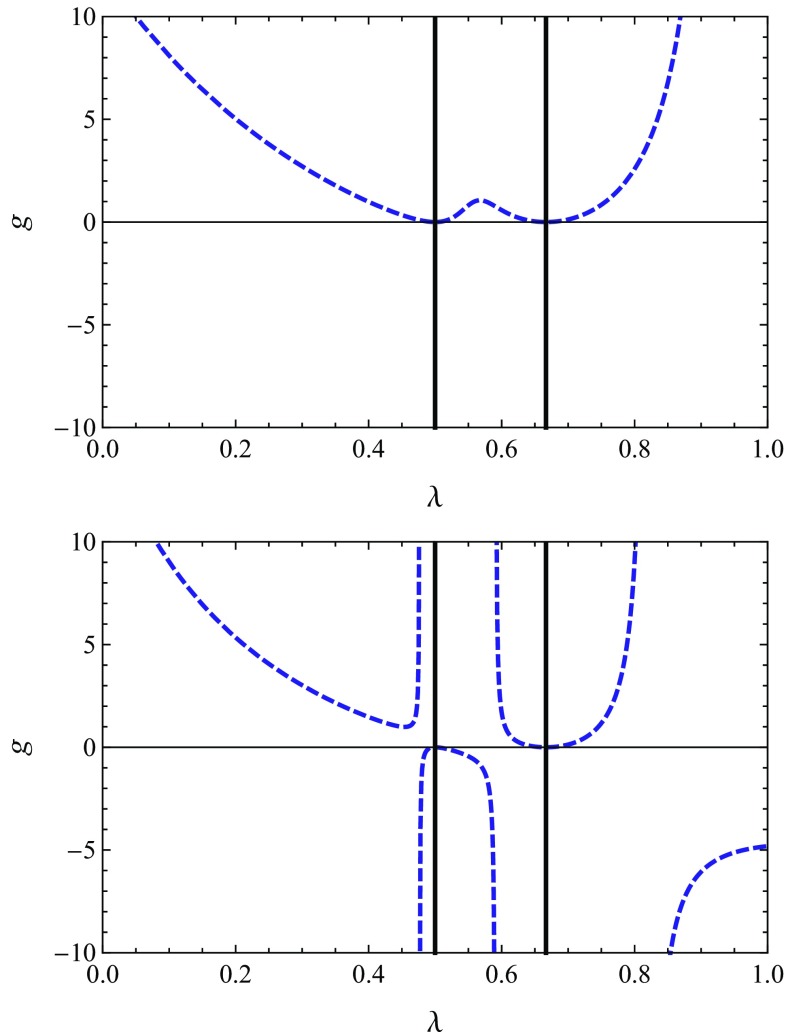
Singular loci of the beta functions (31) for the Type I, linear (*left*) and Type II$_0$, linear (*right*) setup in $d=3$. The *black*, *solid lines* show the fixed singularities (37) while the divergence of $\eta$ is given by the *dashed blue line*. The two diagrams illustrate the two prototypical cases where the fixed singularity is screened (*left*) or unscreened (*right*) by $\eta ^\mathrm{sing}$

The singular lines (37) and (38) are shown in Fig. 1. At this point it is useful to distinguish between the two qualitatively different scenarios.

Focusing on the region where $g>0$ one inevitably encounters a singular line when moving to positive values of $\lambda$. In the first setting, this singular locus is associated with a divergence of the anomalous dimension $\eta$ given by (38). The prototypical singularity structure for this case is shown in the left diagram of Fig. 1. It is realized for the exponential parametrization ($\alpha =1$) and the linear parametrization ($\alpha = 0$) combined with a Type I and Type II$_2$ regularization scheme. In the second setting the locus $\eta ^\mathrm{sing}$ has a pole located before the first fixed singularity. As a consequence the line $\lambda _1^\mathrm{sing}$ is not entirely shielded by the divergence of $\eta$. This scenario is realized for the linear parametrization $\alpha = 0$ with the Type II$_0$ and Type II$_1$ regulator. It will be shown below that the different singularity structures lead to qualitatively different low-energy behaviors of the RG flow in the region $\lambda > 0$.

Subsequently, we analyze the fixed point structure of the beta functions. At a fixed point $(g_*, \lambda _*)$ the beta functions vanish by definition

39 $$\begin{aligned} \beta _g(g_*,\lambda _*) = 0, \quad \beta _\lambda (g_*,\lambda _*) = 0. \end{aligned}$$

In the vicinity of a fixed point the properties of the RG flow can be studied by linearizing the beta functions. The dynamics of the linearized system is encoded in the stability matrix $\mathbf{B}_{ij} = \partial _{g_j} \beta _{g_i}|_{g = g_*}$. The stability coefficients $\theta$ are defined as minus the eigenvalues of $\mathbf{B}_{ij}$. For eigendirections with $\theta > 0$ the solutions are dragged into the fixed point for $k \rightarrow \infty$ while eigendirections with $\theta < 0$ repel the flow in this limit.

**Table 2:** NGFPs of the beta functions (31) evaluated for the linear split ($\alpha = 0$) and the exponential split ($\alpha = 1$) and regulators of Type I and Type II, respectively. The NGFP obtained from the optimization procedure shown in Fig. 2 is listed with the label “optimized”

Fluctuations	Regulator	$g_*$	$\lambda _*$	$g_* \lambda _*$	$\theta _{1,2}$	
Linear	Type I	0.901	0.222	0.200	$1.432 \pm 2.586 i$	
		−	−	−	−	−
	Type $\mathrm {II}_0$	0.896	0.203	0.182	$1.545 \pm 2.032 i$	
		0.342	0.438	0.150	2.774	$-23.89$
	Type $\mathrm {II}_1$	0.879	0.182	0.160	$1.765 \pm 1.787 i$	
		0.510	0.400	0.204	3.016	$-13.28$
	Type $\mathrm {II}_2$	0.901	0.222	0.200	$1.329 \pm 2.332 i$	
		−	$$ -$$	−	−	−
	Optimized	0.900	0.230	0.207	$1.414 \pm 2.941 i$	
		−	$$ -$$	−	−	−
Exponential	Type I	1.049	0.249	0.262	$0.444 \pm 4.041 i$	
	Type $\mathrm {II}_0$	1.050	0.249	0.261	$0.342 \pm 3.855 i$	
	Type $\mathrm {II}_2$	1.050	0.249	0.261	$0.342 \pm 3.855 i$	

**Fig. 2:**
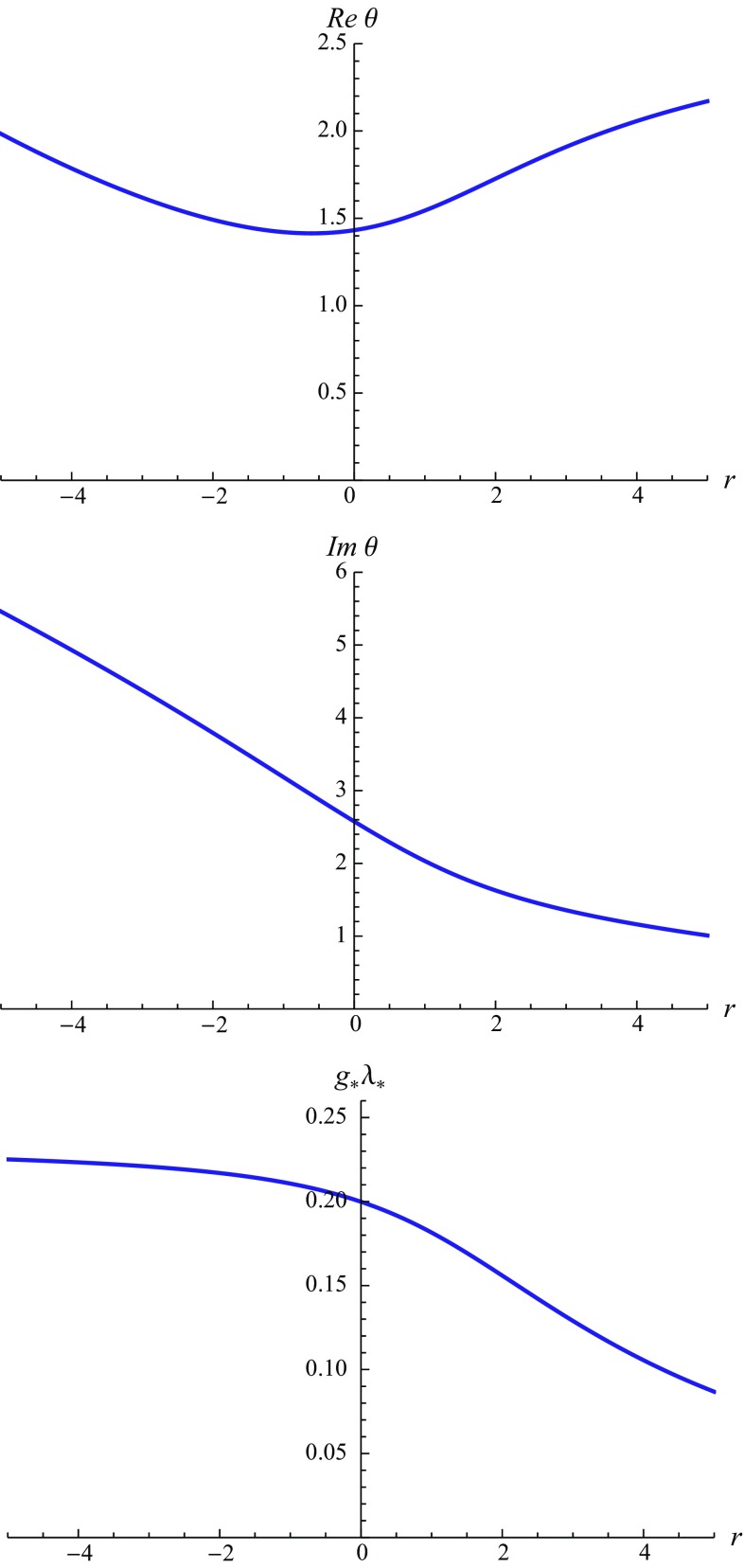
Illustration of the *r*-dependence of the stability coefficients and universal product $g_* \lambda _*$ of the NGFP found for the linear split ($\alpha = 0$). The real part of $\theta$ possesses a minimum for $r_\mathrm{opt}^\mathrm{linear} = -0.605$ while Im$\theta$ and $g_* \lambda _*$ decrease monotonically for increasing *r*

All implementations of the beta functions possess a Gaussian fixed point (GFP) located in the origin. This fixed point corresponds to the free theory and its critical exponents are given by the mass dimension of the dimensionful couplings. Besides the GFP the beta functions also possess non-Gaussian fixed points (NGFPs). Limiting to the physically interesting region with $g >0$ located to the left of the first singular loci, a list of the NGFPs, including their position and stability coefficients, is given in Table 2. For the linear split ($\alpha =0$) all regulators give rise to a NGFP with located at $g> 0,\lambda > 0$. The complex stability coefficients with positive real part indicate that this fixed point is a spiraling attractor which captures the RG flow in its vicinity as $k \rightarrow \infty$. In addition the Type II regulator may give rise to a second NGFP. This fixed point is a saddle point possessing one attractive and one repulsive eigendirection. Notably, the cases which possess this second fixed point coincide with the ones where $\eta ^\mathrm{sing}$ does not screen the singular line $\lambda _1^\mathrm{sing}$, cf. Fig. 1.

The exponential split ($\alpha = 1$) gives rise to a similar picture. In this case the specific structure of the off-diagonal contributions implies that the Type II$_0$ and Type II$_2$ regulators actually coincide, giving rise to the same beta functions. Also this case gives rise to a NGFP with complex critical exponents which acts as a UV-attractor of the flow. The distinct critical exponents accompanying the spiraling NGFPs seen in the linear and exponential setting strongly support that they correspond to two distinct universality classes.^4^ The Type II$_1$ case does not support a NGFP in the physically interesting region. This indicates that the system acts rather sensitive to a change of the eigenvalue spectrum in the scalar $\hat{N}$–*h* sector. Thus we will limit our further considerations to the case $q_\mathrm{non-diag} = 0$.

At this stage, it is natural to ask if there is a preferred value for the parameter *r*. Since a change in the regularization procedure should not affect physical quantities, a natural selection criterion for *r* is to minimize the sensitivity of these quantities with respect to this parameter. This optimization procedure [83–86] may then be used to find a “best value” for the parameter *r*. Within the present computation natural candidates for investigating the *r*-dependence are the stability coefficients and the universal product $g_* \lambda _*$. For a linear split their *r*-dependence is displayed in Fig. 2. While Im$\theta$ and $g_*\lambda _*$ are monotonically decreasing as *r* increases, Re$\theta$ develops a minimum located at $r_\mathrm{opt}^\mathrm{linear} = -0.605$. The corresponding values for the position and stability coefficients of the NGFP are listed in Table 2. A comparison among the characteristic properties of the NGFP reveals that the “optimized values” turn out to be very close to the fixed point seen in the Type I regularization scheme. For the exponential split all physical quantities are monotonic functions of *r*. Thus in this case the principle of minimal sensitivity does not identify a preferred value for *r*.

**Fig. 3:**
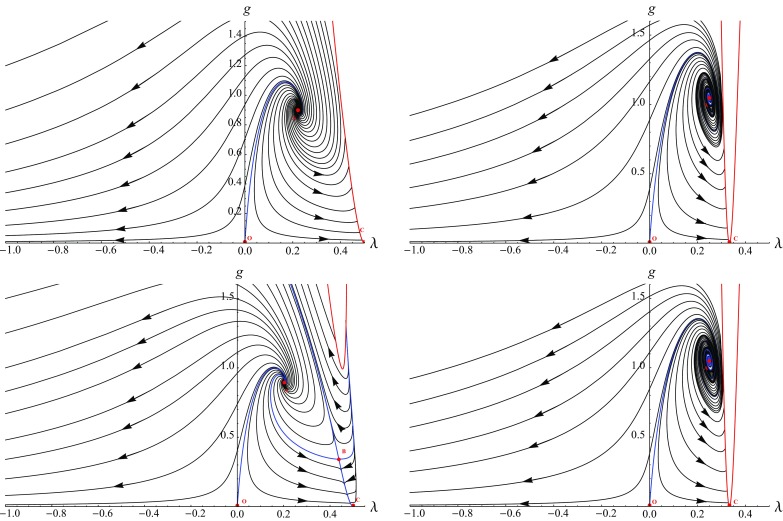
Phase diagrams obtained from integrating the beta functions (29) for a Type I regulator ($r=0$, *top line*) and a Type II$_0$ regulator ($r=1$, *bottom line*). The *first column* gives the result for a linear split of the spatial metric ($\alpha = 0$) while the *second column* corresponds to an exponential split ($\alpha = 1$). All flows possess a NGFP providing the UV-completion of the RG trajectories

Based on the rather detailed discussion of their fixed points and singularity structure, it is rather straightforward to obtain the phase diagrams resulting from integrating the flow equations (29) numerically. Our focus is on the physically interesting region where Newton’s coupling is positive and containing the GFP. The resulting flows are shown in Fig. 3. The left and right column display the results obtained with a linear ($\alpha = 0$) and exponential split ($\alpha = 1$), respectively. The top row stems from a Type I regulator while the bottom row uses the Type II$_0$ regularization scheme. In all cases the GFP is marked with *O* while the non-Gaussian UV attractor carries the label *A*. The red lines mark the singular loci $\eta ^\mathrm{sing}$, Eq. (38), while the blue lines connect the fixed points. All arrows point towards lower RG scales, i.e. in the direction of integrating out modes.

As expected from the results given in Table 2, the flow shows qualitative differences depending on whether the fixed point structure also contains the saddle point *B* (Type II$_0$, linear) or just the NGFP *A* (Type I, linear; exponential split). In the latter case, the phase diagram is dominated by the interplay of the GFP and NGFP. The NGFP controls the high-energy ($k \rightarrow \infty$) limit of all trajectories. Lowering the RG scale, the trajectories undergo a crossover to the GFP. In the vicinity of the GFP the trajectories develop a “classical regime” where the dimensionful couplings are almost independent of the RG scale *k*. Following [35], the solutions are classified according to the value of the cosmological constant in this regime: trajectories located to the left and the right of the blue separatrix give rise to a negative and positive value and are termed Type Ia and Type IIIa, respectively. The blue line separating these phases has a vanishing infrared value of $\Lambda _k$. The trajectories with $\Lambda _0 \le 0$ are complete in the sense that they are well defined on the entire interval $k \in [0,\infty ]$. The trajectories with a positive cosmological constant terminate at $\eta ^\mathrm{sing}$ at a finite value *k*. The presence of the saddle point *B* modifies this very last stage of the RG evolution. In this case the singularity $\eta ^\mathrm{sing}$ is lifted and replaced by a RG trajectory connecting the fixed point *B* and the quasi-fixed point *C* located at $(\lambda ,g) = (1/2,0)$. The flow then follows this line and is dynamically driven into the quasi-fixed point which provides the IR-completion of these trajectories.

**Fig. 4:**
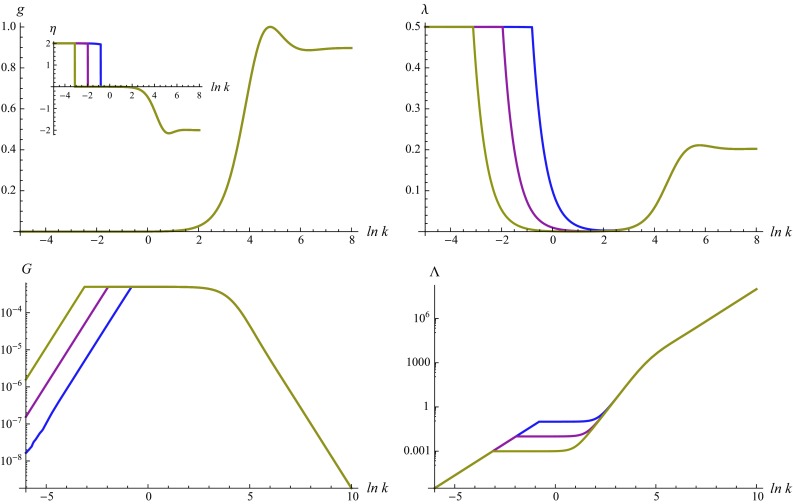
Sample RG trajectories obtained from solving the flow equations for $\alpha = 0$ and a Type II$_0$ regulator for the initial conditions $g_\mathrm{init} = 0.0005$ and $\lambda _\mathrm{init} = 10^{-1}$ (*blue line*), $\lambda _\mathrm{init} = 10^{-2}$ (*magenta line*), and $\lambda _\mathrm{init} = 10^{-3}$ (*gold line*). For $k \rightarrow \infty$ the trajectories are governed by the NGFP. At intermediate scales one obtains a classical regime where the dimensionful $G_k$ and $\Lambda _k$ are independent of *k*. Once $\lambda \lesssim 0.5$ the gravitational instability sets in and drives the dimensionful Newton coupling and cosmological constant to zero dynamically

Figure 4 displays a set of sample trajectories obtained from integrating the beta functions for the linear split with a Type II$_0$ regularization scheme. The top line gives the scale dependence of $g_k$ and $\lambda _k$. They interpolate between the NGFP for $\ln k \gtrsim 6$ and the quasi-fixed point *C* for $\ln k \lesssim 1$. The lower diagrams recasts this flow in terms of the dimensionful couplings $G_k$ and $\Lambda _k$. In this way the three scaling regimes exhibited by the solutions become even more pronounced: in the NGFP regime one has $G_k \propto k^{-2}$ while $\Lambda _k \propto k^2$. The classical regime is situated around $\ln k \approx 0$. Once $\lambda _k$ reaches $\lambda _k \approx 1/2$ the flow enters into a new phase where $G_k$ and $\Lambda _k$ are driven to zero dynamically. The RG trajectories obtained from the other cases shown in Fig. 3 are similar. The only difference consists in the absence of the final low-energy phase. Here the flow terminates in the classical regime at a finite value of *k*.

At this stage, a cautious remark is in order. From the inset in the top-left diagram of Fig. 4 one finds that the new IR phase comes with an anomalous dimension of Newton’s coupling, $\eta _k \approx 2$. This has profound consequences for the regularization procedure. The regulator obtained from the prescription (27) has the structure

40 $$\begin{aligned} \mathcal{R}_k \propto (G_k)^{-1} \, k^2 \, R^{(0)}(\Delta /k^2) \end{aligned}$$

where the profile function $R^{(0)}(\Delta /k^2)$ satisfies

41 $$\begin{aligned} \lim _{k \rightarrow 0} R^{(0)}(\Delta /k^2) = 1. \end{aligned}$$

In general, this asymptotic behavior ensures that the cutoff vanishes as $k\rightarrow 0$. The last property fails, however, if $G_k \propto k^2$ or, equivalently $\eta = 2$, as $k \rightarrow 0$. In this case the *k*-dependence of Newton’s coupling cancels the $k^2$ term and the cutoff $\mathcal{R}_k$ remains finite as $k \rightarrow 0$. As a result, a flow approaching the quasi-fixed point *C* may not integrate out all fluctuation modes, even though the limit $k \rightarrow 0$ is well defined. Of course, all other phases displayed in Fig. 4 are unaffected by this peculiarity.

## Discussion and outlook

In this work we have studied the gravitational renormalization group (RG) flow in the Arnowitt–Deser–Misner (ADM) formalism, utilizing backgrounds with a topology $S^1 \times S^d$. This investigation is mainly motivated through the Causal Dynamical Triangulation (CDT) program where this particular topology has been used extensively in order to study properties of the gravitational partition sum through Monte Carlo simulations. The detailed results reported in the main part of the manuscript and collected in the appendices provide an important stepping stone for comparing properties of the quantum spacetimes arising within the RG and CDT framework.

Our analysis focused on the scale dependence of the (background) Newton’s coupling and cosmological constant obtained from a projection of the functional renormalization group equation (10). The flow possesses a non-Gaussian fixed point (NGFP) suitable for rendering the theory asymptotically safe. The existence of this fixed point is robust with respect to changing the parametrization of the gravitational fluctuations and regularization procedure. The results summarized in Table 2 suggest that the NGFPs obtained from a linear and exponential split of the metric fluctuations belong to two different universality classes. The phase diagrams collected in Fig. 3 show that the flow emanating from the NGFP is connected to a classical regime where Newton’s coupling and the cosmological constant are essentially independent of the renormalization group scale. For specific choices of the regularization schemes, the NGFP is supplemented by a second fixed point solution constituting a saddle point in the *g*–$\lambda$ plane. The interplay of the two fixed points alters the singularity structure of the beta functions which has profound consequences for the infrared behavior of the flow; see Fig. 4.

The results obtained in this work are complementary to the ones reported in [1, 2] which use a very similar construction on a background topology $S^1 \times T^d$. At the geometrical level, the key difference in these two classes of backgrounds is that $S^1 \times S^d$ possesses a Killing vector in the (Euclidean) time-direction which permits a Wick rotation to Lorentzian time without obtaining complex background geometries. At the level of the flow equation, the two projection schemes construct the flow of Newton’s coupling based on two different interaction monomials: the $S^1 \times T^d$ background uses the kinetic terms for the gravitational fluctuations while $S^1 \times S^d$ uses a potential term build from the intrinsic curvature. At the classical Einstein–Hilbert action the relative coefficients of these terms are fixed by the diffeomorphism invariance. At the quantum level it is highly encouraging that the fixed point structure and phase diagrams resulting from these two projection schemes are almost identical.

At this stage constructing gravitational RG flows within the ADM formalism has achieved a similar robustness as the one encountered in comparable computations using a covariant parametrization of the metric fluctuations [34–39], at least at the background level. From the conceptual point of view, it is clear that the background field formalism based on the ADM decomposition gives rise a natural parametrization of the metric fluctuations. This parametrization is related to the one used in the covariant linear split in a non-linear way [2]. In particular, combining the ADM-split and the exponential parametrization of the fluctuations on the spatial slices ensures that the signatures of the time part and spatial part of the physical metric are independently conserved when quantum fluctuations are taken into account. In this sense, the ADM formalism provides a more refined version of the exponential parametrization recently investigated in [63, 64]. An interesting consequence associated with the different parametrization schemes for the metric fluctuations is that it may shift contributions of the RG flow from background vertices to vertices containing fluctuation fields. It would be very interesting to investigate this effect in approximations of the flow equation which also takes vertices containing the fluctuation fields into account. We hope to come back to this point in the future.

The beta functions (31) possess a “gravitational instability” associated with the singular line (37). In Ref. [81], it has been suggested that this type of instability could provide a dynamical solution for the cosmological constant problem through strong RG effects in the infrared. In Fig. 4 we demonstrated that this mechanism may also work in the context of pure gravity. Our general analysis identified two possible caveats to this scenario. First, the gravitational instability may be shielded by a diverging anomalous dimension. This scenario is realized by the flows displayed in the top-left, top-right, and bottom-right diagram of Fig. 3. Second, the anomalous dimension may acquire a value for which the implementation of the Wilsonian RG procedure requires a modification of the (standard) regularization scheme. It would be very interesting to see if dilaton gravity, where Newton’s coupling is generated dynamically, resolves these difficulties in a natural way.

## Appendix A: The background geometry $S^1 \times S^d$

Throughout this work we simplify the computation by choosing a background geometry with topology $S^1 \times S^d$. The size of $S^1$ is kept fixed while the $S^d$ factor denotes a one-parameter family of spheres with arbitrary but fixed radius. Keeping the radius of $S^d$ as a free parameter allows one to disentangle the volume term from an interaction term build from the intrinsic curvature scalar. Thus the background is sufficiently complex for distinguishing the two interaction monomials appearing on the left-hand-side of the projected flow equation.

**Table 3:** Heat-kernel coefficients for fields with differential constraints on the product manifold $S^1 \times S^d$. The *labels* indicate that the resulting $a_i$ have been computed for spacetime scalars (S), spatial vectors (V), spatial, symmetric tensors (T), transverse spatial vectors (TV), and symmetric, transverse-traceless spatial tensors (TTT), respectively

	S	V	T	TV	TTT
$a_0$	1	*d*	$\tfrac{1}{2} d(d+1)$	$d-1$	$\tfrac{1}{2} (d+1)(d-2)$
$a_2$	$\tfrac{1-6q}{6}$	$\tfrac{(1-6q)d}{6}$	$\tfrac{(1-6q) d (d+1)}{12}$	$\tfrac{(d+2)(d-3)+6 \delta _{d,2}}{6d}- q(d-1)$	$\tfrac{(d+1)(d+2)(d-5+3\delta _{d,2})}{12(d-1)}- q \tfrac{(d-2)(d+1)}{2}$

In terms of the $D=d+1$-dimensional geometry, the background metric is given by $\bar{g}_{\mu \nu } = \mathrm{diag}[1, \bar{\sigma }_{ij}]$, where $\bar{\sigma }_{ij}$ denotes the metric on the *d*-sphere. Since $S^d$ is a maximally symmetric space, the Riemann tensor $\bar{R}_{ijkl}$ and the Ricci tensor $\bar{R}_{ij}$ constructed from $\bar{\sigma }_{ij}$ can be related to the Ricci scalar $\bar{R}$ by

A.1 $$\begin{aligned} \bar{R}_{ijkl}= & {} \frac{\bar{R}}{d(d-1)} \, \left( \bar{\sigma }_{ik} \, \bar{\sigma }_{jl} - \bar{\sigma }_{il} \, \bar{\sigma }_{jk} \right) , \nonumber \\ \bar{R}_{ij}= & {} \frac{1}{d} \, \bar{\sigma }_{ij} \, \bar{R}. \end{aligned}$$

In addition the Ricci scalar is covariantly constant, $\bar{D}_i \bar{R}= 0$.

A comparison with the decomposition (3) then indicates that the values of the background ADM fields are given by

A.2 $$\begin{aligned} \bar{N}(\tau ,y) = 1, \quad \bar{N}_i(\tau ,y) = 0, \quad \bar{\sigma }_{ij}(\tau ,y) = \bar{\sigma }_{ij}(y). \end{aligned}$$

The product nature of the background entails that $\bar{\sigma }_{ij}$ is independent of $\tau$ so that derivatives with respect to $\tau$ can be commuted freely with $\bar{\sigma }_{ij}$. Moreover, the Laplacians of the background spacetime, $\Delta \equiv - \bar{g}^{\mu \nu } \bar{D}_\mu \bar{D}_\nu$, and on the spatial slices, $- \bar{\sigma }^{ij} \bar{D}_i \bar{D}_j$, are related by

A.3 $$\begin{aligned} \Delta = - \partial _\tau ^2 - \bar{\sigma }^{ij} \bar{D}_i \bar{D}_j. \end{aligned}$$

This identity allows one to express the differential operators arising in the second variation of $\Gamma _k$ to differential operators constructed from the spacetime metric.

Upon implementing a suitable gauge fixing, all differential operators entering in the right-hand-side of the flow equation can be combined into $\Delta$. The resulting traces can then be evaluated using standard heat-kernel techniques [87, 88]. For the present calculation it suffices to keep track of terms containing at most two covariant derivatives. For a coarse-graining operator of the form

A.4 $$\begin{aligned} \Box = \Delta + q \, \bar{R}, \end{aligned}$$

with *q* being a numerical coefficient, the relevant terms in the expansion are given by

A.5 $$\begin{aligned} \mathrm{Tr}_i \, e^{-s \Box } = \frac{1}{(4 \pi s)^{D/2}} \int \mathrm{{d}}\tau \mathrm{{d}}^dy \bar{N}\sqrt{\bar{\sigma }} \left[ a_0 + a_2 \, s \, \bar{R}+ \cdots \right] . \end{aligned}$$

The terms indicated by the dots do not contributing to the present approximation. The heat-kernel coefficients $a_i$ depend on the index structure of the field and have been given, e.g., in [34]. The result is tabulated in Table 3. The terms proportional to $\delta _{2,d}$ arise from eigenmodes of the Laplacian which do not contribute to the fields appearing on the left-hand-side of the decompositions (21) and (22).

## Appendix B: Evaluation of the second variations

This appendix summarizes the construction of the Hessian $\Gamma _k^{(2)}$ on a background $S^1 \times S^d$.

### Appendix B.1: The gravitational sector

We start by expanding the gravitational action (11) to second order in the fluctuation fields. In order to facilitate the computation, the Einstein–Hilbert action (11) is decomposed into four interaction monomials

B.6 $$\begin{aligned} I_1\equiv & {} \int \mathrm{{d}}\tau \mathrm{{d}}^dy \, N \sqrt{\sigma } \, K_{ij} K^{ij}, \nonumber \\ I_2\equiv & {} \int \mathrm{{d}}\tau \mathrm{{d}}^dy \, N \sqrt{\sigma } \, K^2, \nonumber \\ I_3\equiv & {} \int \mathrm{{d}}\tau \mathrm{{d}}^dy \, N \sqrt{\sigma } \, R, \nonumber \\ I_4\equiv & {} \int \mathrm{{d}}\tau \mathrm{{d}}^dy \, N \sqrt{\sigma }. \end{aligned}$$

Moreover, we use the shorthand notations

B.7 $$\begin{aligned} \int _y \equiv \int \mathrm{{d}}\tau \, \mathrm{{d}}^dy \, \sqrt{\bar{\sigma }}, \quad \text{ and } \quad \bar{D}^2 \equiv \bar{\sigma }^{ij} \bar{D}_i \bar{D}_j, \end{aligned}$$

to more easily treat our formulas. The expansion of the kinetic terms $I_1$ and $I_2$ around the background (13) yields the following terms quadratic in the fluctuation fields:

B.8 $$\begin{aligned} \delta ^2 I_1 \!= & {} \! \! \int _y \Big [\tfrac{1}{2} ( \partial _{\tau } \hat{\sigma }_{ij} ) ( \partial _{\tau } \hat{\sigma }^{ij} ) - 2 (\bar{D}^i \hat{N}^j)(\partial _{\tau } \hat{\sigma }_{ij}) \nonumber \\&+\, (\bar{D}_i \hat{N}_j + \bar{D}_j \hat{N}_i) (\bar{D}^i \hat{N}^j) \Big ], \; \; \end{aligned}$$

B.9 $$\begin{aligned} \delta ^2 I_2 \!= & {} \! \! \int _y \left[ \tfrac{1}{2} ( \partial _{\tau } \hat{\sigma } )^2 - 2 (\bar{D}^i \hat{N}_i) (\partial _{\tau } \hat{\sigma }) + 2( \bar{D}^i \hat{N}_i )^2 \right] . \end{aligned}$$

The result is independent of the decomposition of the spatial metric in terms of background and fluctuation fields (6): the parameter $\alpha$ encoding the difference between the linear and exponential split does not enter these expressions. The expansion of the potential terms $I_3$ and $I_4$ is given by

B.10 $$\begin{aligned} \delta ^2 I_3= & {} \! \! \int _y \, \Big [ \tfrac{1}{2} \hat{\sigma }_{ij} \bar{D}^2 \hat{\sigma }^{ij} - \hat{\sigma }^{ij} \bar{D}_j \bar{D}_k \hat{\sigma }^k_i \nonumber \\&+\, (2\hat{N} + \hat{\sigma }) \bar{D}_i \bar{D}_j \hat{\sigma }^{ij} - (2\hat{N} + \tfrac{1}{2}\hat{\sigma }) \bar{D}^2 \hat{\sigma } \qquad \quad \nonumber \\&+\, \bar{R} \, \Big ( \tfrac{d-2}{d} \hat{N}\hat{\sigma } + \tfrac{d^2-5d+8}{4d(d-1)} \hat{\sigma }^2 \nonumber \\&-\, \tfrac{d^2-3d+4}{2d(d-1)} \hat{\sigma }^{ij}\hat{\sigma }_{ij} + \alpha \, \tfrac{d-2}{4d} \, \hat{\sigma }^{ij}\hat{\sigma }_{ij} \Big ) \Big ] \, ,\end{aligned}$$

B.11 $$\begin{aligned} \delta ^2 I_4= & {} \! \! \int _y \, \left[ \hat{N}\hat{\sigma } + \tfrac{1}{4} \hat{\sigma }^2 - \tfrac{1}{2} \left( 1 - \tfrac{\alpha }{2} \right) \hat{\sigma }^{ij}\hat{\sigma }_{ij} \right] . \end{aligned}$$

The $\alpha$-dependence of $\delta ^2 I_3$ and $\delta ^2 I_4$ indicates that this sector receives extra terms when the exponential split is implemented.

In order to facilitate the next steps of the computation, we perform a transverse-traceless decomposition of the fluctuation fields with respect to the background. For fluctuations in the shift vector $\hat{N}_i$ we use

B.12 $$\begin{aligned} \hat{N}_i = u_i + \bar{D}_i B, \quad \bar{D}^i u_i = 0, \end{aligned}$$

where the constraint ensures that $u_i$ is transverse. Analogously, the fluctuations of the spatial metric are decomposed in a transverse-traceless tensor $h_{ij}$, a transverse vector $v_i$ and two scalars $\psi$ and *h*,

B.13 $$\begin{aligned} \hat{\sigma }_{ij} = h_{ij} + \bar{D}_i v_j + \bar{D}_j v_i + \left( \bar{D}_i \bar{D}_j-\tfrac{1}{d}\, \bar{\sigma }_{ij} \, \bar{D}^2\right) \psi + \tfrac{1}{d} \, \bar{\sigma }_{ij}h \end{aligned}$$

satisfying

B.14 $$\begin{aligned} \bar{D}^i h_{ij} = 0, \quad \bar{\sigma }^{ij} h_{ij} = 0, \quad \bar{D}^i v_i = 0, \end{aligned}$$

together with $\bar{\sigma }^{ij} \hat{\sigma }_{ij} \equiv \hat{\sigma }= h$. This change of integration variables is accompanied by non-trivial Jacobians. Their form can be deduced by evaluating the scalar products

B.15 $$\begin{aligned} \int _y \hat{N}_i \hat{N}^i= & {} \int _y \Big [ u_i u^i - B\bar{D}^2 B \Big ], \nonumber \\ \int _y \hat{\sigma }_{ij} \hat{\sigma }^{ij}= & {} \int _y \Big [ h_{ij}h^{ij} - 2 v_i \left[ \bar{D}^2 + \tfrac{\bar{R}}{d} \right] v^i \nonumber \\&+\, \tfrac{d-1}{d} \, \psi \left[ \bar{D}^2 \left( \bar{D}^2+ \tfrac{\bar{R}}{(d-1)} \right) \right] \psi +\tfrac{1}{d}h^2 \Big ].\nonumber \\ \end{aligned}$$

These Jacobians can then be taken into account by an additional field redefinition

B.16 $$\begin{aligned} B&\mapsto \big (-\bar{D}^2\big )^{-1/2}B, \nonumber \\ v_i&\mapsto \big (-\bar{D}^2 - \tfrac{\bar{R}}{d}\big )^{-1/2} v_i, \nonumber \\ \psi&\mapsto \big (-\bar{D}^2\big )^{-1/2} \big (-\bar{D}^2 - \tfrac{\bar{R}}{d-1}\big )^{-1/2} \psi . \end{aligned}$$

This rescaling also ensures that all fields appearing in the decompositions have the same dimensionality. With a slight abuse in notation, we will work with the rescaled fields in the sequel.

Implementing the transverse-traceless decomposition in the kinetic terms and taking into account the rescaling (B.16) yields

B.17 $$\begin{aligned} \delta ^2 I_1= & {} \! \! \int _y \Big [ -\tfrac{1}{2} h_{ij} \, \partial _{\tau }^2 \, h^{ij} - v_i \, \partial _{\tau }^2 \, v^i + u_i \left[ -\bar{D}^2 -\tfrac{\bar{R}}{d}\right] u^i\nonumber \\&+\, 2B\left[ -\bar{D}^2-\tfrac{\bar{R}}{d}\right] B - \tfrac{d-1}{2d} \psi \, \partial _{\tau }^2 \, \psi - \tfrac{1}{2d} \, h \, \partial _{\tau }^2 \, h \nonumber \\&-\, 2u_i \, \partial _{\tau }\left[ -\bar{D}^2 -\tfrac{R}{d}\right] ^{1/2} v^i \nonumber \\&-\, \tfrac{2(d-1)}{d} B\partial _{\tau }\left[ -\bar{D}^2-\tfrac{\bar{R}}{d-1}\right] ^{1/2}\psi \nonumber \\&+\, \tfrac{2}{d} \, B \, \partial _{\tau } \left[ -\bar{D}^2\right] ^{1/2} \, h \Big ] \, ,\end{aligned}$$

B.18 $$\begin{aligned} \delta ^2 I_2= & {} \! \! \int _y \Big [ -\tfrac{1}{2}h\partial _{\tau }^2 h - 2B\bar{D}^2 B \nonumber \\&+\, 2B\, \partial _{\tau } \left[ -\bar{D}^2\right] ^{1/2} \, h \Big ]. \end{aligned}$$

The potential terms become

B.19 $$\begin{aligned} \delta ^2 I_3= & {} \! \! \int _y \Big [\tfrac{1}{2}h_{ij}\left[ \bar{D}^2 - \tfrac{d^2-3d+4}{d(d-1)} \bar{R}\right] h^{ij}\nonumber \\&-\, \tfrac{d-2}{d} \, \bar{R}\, v_i v^i + \tfrac{(d-2)(d-1)}{2d^2} \psi \left[ -\bar{D}^2 - \bar{R}\right] \psi \nonumber \\&+\, \tfrac{(d-2)(d-1)}{2d^2} h\left[ -\bar{D}^2+\tfrac{d-4}{2(d-1)}\bar{R}\right] h \nonumber \\&+\, \tfrac{(d-2)(d-1)}{d^2} \, \psi \left[ \bar{D}^2\left( \bar{D}^2+\tfrac{\bar{R}}{d-1}\right) \right] ^{1/2} h \nonumber \\&- \, \hat{N}\left[ \tfrac{2d-2}{d}\bar{D}^2 -\tfrac{d-2}{d} \bar{R}\right] h \nonumber \\&+\, \tfrac{2(d-1)}{d} \, \hat{N}\left[ \bar{D}^2\left( \bar{D}^2 + \tfrac{\bar{R}}{d-1}\right) \right] ^{1/2} \psi \nonumber \\&+\, \tfrac{d-2}{4d} \, \alpha \, \bar{R}\left( h_{ij}h^{ij} + 2 \, v_i v^i + \tfrac{d-1}{d} \, \psi ^2 + \tfrac{1}{d} \, h^2 \right) \Big ],\nonumber \\ \end{aligned}$$

B.20 $$\begin{aligned} \delta ^2 I_4= & {} \! \! \int _y \Big [ \hat{N}h + \tfrac{1}{4}h^2 - \tfrac{1}{2} \left( 1 - \tfrac{\alpha }{2} \right) \nonumber \\&\times \, \left( h_{ij}h^{ij} + 2 \, v_i v^i + \tfrac{d-1}{d} \, \psi ^2 + \tfrac{1}{d} \, h^2 \right) \Big ]. \end{aligned}$$

Based on these building blocks, the part of $\Gamma _k^\mathrm{grav}$ quadratic in the fluctuation fields can be obtained:

B.21 $$\begin{aligned} \delta ^{2}&\Gamma _k^\mathrm{grav} = \frac{1}{16 \pi G_k} \left( \delta ^2 I_1 - \delta ^2 I_2 - \delta ^2 I_3 + 2 \Lambda _k \, \delta ^2 I_4 \right) . \end{aligned}$$

It is an intriguing observation that the kinetic terms do not depend on the specific decomposition of the spatial metric into background and fluctuation fields. The parameter $\alpha$ encoding the contributions from the exponential decomposition appears in the potential terms only.

### Appendix B.2: The gauge-fixing sector

Inspecting Eqs. (B.18)–(B.19) more closely, one recognizes that the spatial vector field $v^i$ has a rather peculiar dispersion relation which does not contain spatial derivatives. This feature persists if one adopts proper time gauge where the fluctuations in the lapse function and shift vector are zero. The gauge fixing (18) cures this problem. This is demonstrated as follows.

Following the procedure of defining building blocks, it is natural to express the gauge-fixing action in terms of the two expressions

B.22 $$\begin{aligned} \delta ^2 I_5 = \int _x F^2,\quad \delta ^2 I_6 = \int _x F_i \, \bar{\sigma }^{ij} \, F_j. \end{aligned}$$

The functionals *F* and $F_i$ are linear in the fluctuation fields, so that these building blocks are quadratic in the fluctuations. This feature is indicated by labeling the blocks by $\delta ^2 I_5$ and $\delta ^2 I_6$. Substituting the field decompositions (B.12) and (B.13) and invoking the rescaling (B.16) the contributions (B.22) are

B.23 $$\begin{aligned} \delta ^2 I_5= & {} \! \! \int _y \Big [\hat{N}\partial _{\tau }^2 h + B \, \partial _{\tau }\left[ -\bar{D}^2 \right] ^{1/2} \, \big ( h - 2 \hat{N}\big ) \\ \nonumber&-\, \hat{N}\partial _{\tau }^2\hat{N} - B\bar{D}^2 B - \tfrac{1}{4}h\partial _{\tau }^2h \Big ] \, \\ \nonumber \end{aligned}$$

and

B.24 $$\begin{aligned} \delta ^2 I_6= & {} \! \! \int _y \Big [ v_i\left[ -\bar{D}^2-\tfrac{\bar{R}}{d}\right] v^i - u_i \partial _{\tau }^2 u^i \end{aligned}$$

B.25 $$\begin{aligned}&+\,2 \, u_i \, \partial _{\tau }\left[ -\bar{D}^2 -\tfrac{\bar{R}}{d}\right] ^{1/2} v^i - \tfrac{(d-2)^2}{4d^2} h \, \bar{D}^2 \, h \nonumber \\&-\, \tfrac{(d-1)^2}{d^2} \, \psi \left[ \bar{D}^2+\tfrac{\bar{R}}{d-1} \right] \psi - B \, \partial _{\tau }^2 \, B \nonumber \\&-\, \hat{N} \, \bar{D}^2 \, \hat{N} + 2B \, \partial _{\tau } \left[ - \bar{D}^2 \right] ^{1/2} \, \hat{N} \nonumber \\&+\, \tfrac{(d-2)(d-1)}{d^2} \psi \, \left[ \bar{D}^2 \left( \bar{D}^2 + \tfrac{\bar{R}}{d-1}\right) \right] ^{1/2} \, h \nonumber \\&+\, \tfrac{2(d-1)}{d} \, B \, \partial _{\tau } \left[ -\bar{D}^2-\tfrac{\bar{R}}{d-1} \right] ^{1/2} \, \psi \nonumber \\&+\, \tfrac{2(d-1)}{d}\psi \left[ \bar{D}^2 \left( \bar{D}^2 + \tfrac{\bar{R}}{d-1}\right) \right] ^{1/2} \hat{N} \nonumber \\&-\, \tfrac{d-2}{d} h \, \partial _{\tau } \left[ - \bar{D}^2 \right] ^{1/2} \, B - \tfrac{d-2}{d} \, h \, \bar{D}^2 \hat{N} \Big ]. \end{aligned}$$

**Table 4:** List of non-zero matrix elements appearing in the Hessian $\Gamma _k^{(2)}$. The gravitational sector is obtained from the combination (B.26) while the ghost contributions given in the last two lines arise from (20). Each off-diagonal entry is accompanied by a suitable entry with the order of the fields reversed

	$$(32 \pi G_k)^{-1} \left[ \Gamma _k^{(2)} \right] _{ab}$$
$h_{ij} h^{ij}$	$\Delta - 2 \left( 1-\tfrac{\alpha }{2}\right) \Lambda _k + \left( \tfrac{d^2-3d+4}{d(d-1)} - \tfrac{d-2}{2d} \, \alpha \right) \bar{R}$
$v_i \, v^i$	$2 \left( \Delta - 2 \left( 1-\tfrac{\alpha }{2}\right) \Lambda _k + \left( \tfrac{d-3}{d} - \tfrac{d-2}{2d} \, \alpha \right) \bar{R}\right)$
$\psi ^2$	$\tfrac{d-1}{d} \left( \Delta - 2 \left( 1-\tfrac{\alpha }{2}\right) \Lambda _k + \left( \tfrac{d-4}{d} - \tfrac{d-2}{2d} \alpha \right) \bar{R}\right)$
$u_i \, u^i$	$2 \left( \Delta - \tfrac{1}{d} \bar{R}\right)$
$B^2$	$2 \left( \Delta - \tfrac{2}{d} \bar{R}\right)$
$h^2$	$- \tfrac{d-2}{2d} \left( \Delta - 2 \Lambda _k + \left( \tfrac{d-4}{d} + \tfrac{\alpha }{d} \right) \bar{R}\right) + \tfrac{\alpha }{d} \, \Lambda _k$
$\hat{N}h$	$- \left( \Delta - 2 \Lambda _k + \tfrac{d-2}{d} \bar{R}\right)$
$\hat{N}^2$	$2 \Delta$
$\bar{c}c$	$(32 \pi G_k) \, \Delta$
$\bar{b}^i b_i$	$(32 \pi G_k) \, \left( \Delta - \tfrac{1}{d} \bar{R}\right)$

The matrix elements appearing in the gravitational sector of the Hessian $\Gamma _k^{(2)}$ can then be read off from the combination

B.26 $$\begin{aligned} \tfrac{1}{2} \delta ^2 \Gamma _k^\mathrm{grav} + \Gamma _k^\mathrm{gauge\ fixing}. \end{aligned}$$

They are summarized in Table 4. Owing to the specific choice of the gauge fixing all differential operators combine into Laplacians of the $D=d+1$-dimensional background manifold (A.3). Based on the Hessian given in Table 4, the regulator $\mathcal{R}_k$ is constructed from the prescription (27). This completes the explicit construction of the traces appearing on the right-hand-side of the flow equation.

## Appendix C: Evaluation of the operator traces

Based on the results collected in Table 4, the operator traces appearing on the right-hand-side of the flow equation can be evaluated by combining standard Mellin-transform techniques, reviewed in [24], with the heat-kernel formula (A.5). We start by giving a master formula for evaluating the operator traces in Appendix C.1 while the explicit results are collected in Appendix C.2.

### Appendix C.1: The master formula

The contributions of the operator traces appearing in the FRGE are conveniently expressed in terms of the dimensionless threshold functions [32]

C.27 $$\begin{aligned} \Phi ^p_n(w)\equiv & {} \frac{1}{\Gamma (n)} \int _0^\infty \mathrm{{d}}z \, z^{n-1} \, \frac{R^{(0)}(z) - z R^{(0)\prime }(z)}{[z+ R^{(0)}(z) + w]^p}, \nonumber \\ \widetilde{\Phi }^p_n(w)\equiv & {} \frac{1}{\Gamma (n)} \int _0^\infty \mathrm{{d}}z \, z^{n-1} \, \frac{R^{(0)}(z)}{[z+ R^{(0)}(z) + w]^p}. \end{aligned}$$

For a cutoff of Litim type [36], $R_k=(k^2-\Delta _s)\,\theta (k^2-\Delta _s)$, to which we resort in the main part of the paper the integrals in the threshold functions can be evaluated analytically

C.28 $$\begin{aligned} \Phi ^p_n(w)= & {} \frac{1}{\Gamma (n+1)} \, \frac{1}{(1+w)^p}, \nonumber \\ \widetilde{\Phi }^p_n(w)= & {} \frac{1}{\Gamma (n+2)} \, \frac{1}{(1+w)^p}. \end{aligned}$$

Apart from the $\hat{N}$–*h* sector, all Hessians given in Table 4 have the structure^5^

C.29 $$\begin{aligned} \mathcal{P}= \left( 32 \pi G_k \right) ^{-s} \left( \Box + w \Lambda _k + c \bar{R}\right) \mathbbm {1} \, , \end{aligned}$$

where $\mathbbm {1}$ is the unit on the internal space and the parameter $s = 0,1$ in the ghost sector and gravitational sector respectively. The contributions of the fluctuation fields to the flow can then be obtained from the following master formula:

C.30 $$\begin{aligned} \mathrm{Tr} \Big [ \mathcal{P}^{-1} \partial _t \mathcal{R}_k \Big ]= & {} \frac{k^D}{(4\pi )^{D/2}} \int _y \Big [ a_0 \left( 2 \, \Phi ^1_{D/2}(\widetilde{w}) - s \, \eta \, \widetilde{\Phi }^1_{D/2}(\widetilde{w}) \right) \nonumber \\&+\, a_2 \left( 2 \, \Phi ^1_{D/2-1}(\widetilde{w}) - s \, \eta \, \widetilde{\Phi }^1_{D/2-1}(\widetilde{w}) \right) \tfrac{\bar{R}}{k^2} \nonumber \\&-\, c \, a_0 \left( 2 \, \Phi ^2_{D/2}(\widetilde{w}) - s \, \eta \, \widetilde{\Phi }^2_{D/2}(\widetilde{w}) \right) \tfrac{\bar{R}}{k^2} \Big ].\nonumber \\ \end{aligned}$$

The heat-kernel coefficients $a_n$ depend on the spin and endomorphism parameter *q* and are tabulated in Table 3. The anomalous dimension $\eta$ is defined in (28) and $\widetilde{w}\equiv w \lambda _k$ is the dimensionless version of the combination $w \Lambda _k$. For a Type II regulator $c=0$ by construction.

### Appendix C.2: Explicit results for the operator traces

Based on the master formula (C.30), it is rather straightforward to find the contributions of the terms listed in Table 4 to the projection of the RG flow.

#### Appendix C.2.1: Diagonal terms in the gravitational sector

The master formula (C.30) indicates that in the gravitational sector the threshold functions always appear in the combination

C.31 $$\begin{aligned} q_n^p(\widetilde{w}) \equiv 2 \, \Phi ^p_{n}(\widetilde{w}) - \eta \, \widetilde{\Phi }^p_{n}(\widetilde{w}). \end{aligned}$$

In the diagonal sector, the argument $\widetilde{w}$ captures the dependence of the traces on the cosmological constant and it is convenient to set

C.32 $$\begin{aligned} \widetilde{w}_d \equiv (\alpha - 2) \, \lambda . \end{aligned}$$

Notably, the value $\alpha = 2$ (which does not correspond to an exponential split) is special since the $\lambda$-dependence of the flow is restricted to the off-diagonal sector. Making use of the master formula (C.30) the contributions of the component fields associated with the spatial metric are

C.33 $$\begin{aligned} \mathrm{Tr}_{h_{ij}h^{ij}}= & {} \tfrac{k^D (d+1)(d-2)}{2(4\pi )^{D/2}}\int _x \Bigl [q^1_{D/2} \nonumber \\&+\, \left( \tfrac{d^2-3d+4}{d(d-1)}-\tfrac{d-2}{2d}\alpha \right) (r-1) \, q^2_{D/2} \, \tfrac{\bar{R}}{k^2}\nonumber \\&+\, \left( \tfrac{(d+2)(d-5+3\delta _{d,2})}{6(d-1)(d-2)}\right) \, q^1_{D/2-1} \, \tfrac{\bar{R}}{k^2} \Bigr ], \nonumber \\&-\, r\left( \tfrac{d^2-3d+4}{d(d-1)}-\tfrac{d-2}{2d}\alpha \right) \, q^1_{D/2-1} \, \tfrac{\bar{R}}{k^2} \Bigr ], \nonumber \\ \mathrm{Tr}_{v_{i}v^{i}}= & {} \tfrac{k^D (d-1)}{(4\pi )^{D/2}}\int _x \Bigl [q^1_{D/2} \nonumber \\&+\, \left( \tfrac{d-3}{d}-\tfrac{d-2}{2d}\alpha \right) \, (r-1) \, q^2_{D/2} \, \tfrac{\bar{R}}{k^2}\nonumber \\&+\, \left( \tfrac{(d+2)(d-3)+6\delta _{d,2}}{6d(d-1)} - r\left( \tfrac{d-3}{d}-\tfrac{d-2}{2d}\alpha \right) \right) \, q^1_{D/2-1} \, \tfrac{\bar{R}}{k^2} \Bigr ], \nonumber \\ \mathrm{Tr}_{\psi \psi }= & {} \tfrac{k^D }{(4\pi )^{D/2}}\int _x \Bigl [q^1_{D/2} \nonumber \\&+\, \left( \tfrac{d-4}{d}-\tfrac{d-2}{2d}\alpha \right) \, (r-1) \, q^2_{D/2} \, \tfrac{\bar{R}}{k^2} \nonumber \\&+\,\left( \tfrac{1}{6} r\left( \tfrac{d-4}{d}-\tfrac{d-2}{2d}\alpha \right) \right) \, q^1_{D/2-1} \, \tfrac{\bar{R}}{k^2} \Bigr ]. \end{aligned}$$

Here the argument of all threshold functions is given by (C.32).

The traces containing fields *B* and $u_i$, arising from the decomposition of the shift vector, do not receive a contribution from the cosmological constant. In this case the threshold functions $q_n^p$ are evaluated at zero argument and the resulting trace contributions read

C.34 $$\begin{aligned} \mathrm{Tr}_{BB}= & {} \tfrac{k^D }{(4\pi )^{D/2}}\int _x \Big [ q^1_{D/2} \nonumber \\&+\;\left( \tfrac{1}{6} + \tfrac{2r}{d} \right) \, q^1_{D/2-1} \, \tfrac{\bar{R}}{k^2} - \tfrac{2}{d} \, (r-1) \, q^2_{D/2} \, \tfrac{\bar{R}}{k^2} \Big ], \nonumber \\ \mathrm{Tr}_{u_{i}u^{i}}= & {} \tfrac{k^D (d-1)}{(4\pi )^{D/2}}\int _x \Bigl [q^1_{D/2} \nonumber \\&+\,\left( \tfrac{(d+2)(d-3)+6\delta _{d,2}}{6d(d-1)} + \tfrac{r}{d}\right) \, q^1_{D/2-1} \, \tfrac{\bar{R}}{k^2} \nonumber \\&-\, \tfrac{1}{d} \, (r-1) \, q^2_{D/2} \, \tfrac{\bar{R}}{k^2} \Bigr ]. \end{aligned}$$

The results (C.33) and (C.34) complete the evaluation of the operator traces appearing in the diagonal part of the gravitational sector.

#### Appendix C.2.2: Off-diagonal terms in the gravitational sector

The final contribution in the gravitational sector originates from the sector spanned by the fluctuations of the lapse function $\hat{N}$ and the conformal modes of the spatial metric *h*. In this sector the fluctuations span a full $2\times 2$ block matrix in field space. Following the Type I regularization procedure, one can construct the cutoff $\mathcal{R}_k$ in this sector and subsequently invert this block matrix in field space. The propagators appearing in this procedure are not of the simple form (C.29) but contain terms quadratic in the spacetime Laplacians. Using the specific properties of the Litim-type regulator (C.28), the result can still be expressed in terms of the threshold functions. For the Type I regulator, the resulting contribution is given by

C.35 $$\begin{aligned} \mathrm{Tr}_s= & {} \tfrac{k^D}{(4\pi )^{D/2}} \int _y \Big [ \left( 2-\tfrac{3d-2+\alpha }{d-1} \lambda \right) \\ \nonumber&\times \,\left( q^1_{D/2}(\widetilde{w}_s) + \tfrac{1}{6} \, q^1_{D/2-1}(\widetilde{w}_s) \, \tfrac{\bar{R}}{k^2} \right) \\ \nonumber&- \tfrac{d-2}{2d(d-1)} \left( \tfrac{3 d^2 - 7 d + 4}{d-1} + \alpha - 8 d \lambda + \tfrac{2d(3d+\alpha )}{d-1} \lambda ^2 \right) \\ \nonumber&\times \, q^2_{D/2}(\widetilde{w}_s) \, \tfrac{\bar{R}}{k^2} \Big ], \end{aligned}$$

where

C.36 $$\begin{aligned} \widetilde{w}_s \equiv - \tfrac{3d-2+\alpha }{d-1} \, \lambda + \tfrac{2d}{d-1} \, \lambda ^2. \end{aligned}$$

The implementation of a Type II regulator in this sector is non-trivial. This complication can be traced back to the feature that, in contrast to the other fields, the terms in the off-diagonal sector come with different relative coefficients between the Laplacians $\Delta$ and the intrinsic curvature $\bar{R}$. Inspecting the structure of $\Gamma _k^{(2)} + \mathcal{R}_k$ reveals that there are two natural candidates for choosing an endomorphism in the regulator

C.37 $$\begin{aligned} \Box _1 =&\, \Delta + \tfrac{d-2}{d} \bar{R}, \quad \Box _2 = \Delta + \tfrac{d-4+\alpha }{d} \bar{R}. \end{aligned}$$

The inclusion of the non-zero endomorphism slightly modifies the contribution of the scalar sector. For the choice $\Box _1$ the trace evaluates to

C.38 $$\begin{aligned} \mathrm{Tr}_s= & {} \tfrac{k^D}{(4\pi )^{D/2}} \int _y \Big [ \left( 2-\tfrac{3d-2+\alpha }{d-1} \lambda \right) \nonumber \\&\times \, \left( q^1_{D/2}(\widetilde{w}_s) -\tfrac{5d-12}{6d} \, q^1_{D/2-1}(\widetilde{w}_s) \, \tfrac{\bar{R}}{k^2} \right) \nonumber \\&+\, \tfrac{d-2}{2d} \Big ( \tfrac{d- \alpha }{d-1} - \tfrac{4 (d-2 + \alpha )}{d-1} \lambda \nonumber \\&+\, \tfrac{2 (2 d^2 - 8 d + 4 + (5d- 4) \alpha + \alpha ^2)}{(d-1)^2} \lambda ^2 \Big ) \, q^2_{D/2}(\widetilde{w}_s) \, \tfrac{\bar{R}}{k^2} \Big ], \end{aligned}$$

while for the second choice, $\Box _2$, one obtains

C.39 $$\begin{aligned} \mathrm{Tr}_s= & {} \tfrac{k^D}{(4\pi )^{D/2}} \int _y \Big [ \left( 2-\tfrac{3d-2+\alpha }{d-1} \lambda \right) \nonumber \\&\times \, \left( q^1_{D/2}(\widetilde{w}_s) -\tfrac{5d-24+6\alpha }{6d} \, q^1_{D/2-1}(\widetilde{w}_s) \, \tfrac{\bar{R}}{k^2} \right) \nonumber \\&+ \, \tfrac{1}{d} \Big ( \tfrac{d^2-10d+8 + (3d - 2) \alpha }{2(d-1)} - 2 \, \tfrac{d^2-10d+8 + (4d - 6) \alpha + \alpha ^2}{d-1} \lambda \nonumber \\&+\, \tfrac{2 d^3 - 22 d^2 + 36d -16 + (10 d^2 - 34 d + 20 ) \alpha + (7 d-8 ) \alpha ^2 + \alpha ^3}{(d-1)^2} \lambda ^2 \Big ) \nonumber \\&\times \, q^2_{D/2}(\widetilde{w}_s) \, \tfrac{\bar{R}}{k^2} \Big ]. \end{aligned}$$

This result completes the evaluation of the operator traces appearing in the gravitational sector.

#### Appendix C.2.3: Ghost contributions

In the ghost sector all threshold functions are evaluated at zero argument and there are no terms containing $\eta$. The explicit contributions of the traces are given by

C.40 $$\begin{aligned} - \mathrm{Tr}_{\bar{c}c}= & {} - \tfrac{k^D}{(4\pi )^{D/2}} \int _y \Big [ 2 \, \Phi ^1_{D/2} + \tfrac{1}{3} \, \Phi ^1_{D/2-1} \, \tfrac{\bar{R}}{k^2} \Big ], \end{aligned}$$

C.41 $$\begin{aligned} - \mathrm{Tr}_{\bar{b}b}= & {} - \tfrac{k^D}{(4\pi )^{D/2}} \int _y \Big [ 2 d \, \Phi ^1_{D/2} + \left( \tfrac{1}{3} d + 2 r \right) \, \Phi ^1_{D/2-1} \, \tfrac{\bar{R}}{k^2} \nonumber \\&+\, 2 \left( 1-r\right) \Phi ^2_{D/2} \, \tfrac{\bar{R}}{k^2} \Big ]. \end{aligned}$$

Together with Eqs. (C.33), (C.34), and (C.35), this result completes the evaluation of all traces appearing on the right-hand-side of the FRGE.

## Footnotes

1 For related work in the context of Euclidean Dynamical Triangulations see [11–14].

2 For selected work on Asymptotic Safety in gravity–matter models see [66–72].

3 For applications of this formalism to Hořava–Lifshitz gravity [77] see [78, 79].

4 This agrees with the conclusion reached in [82] where it was shown that the linear and exponential parametrization lead to Liouville theories with different central charge.
